# Serum miR-331-3p predicts tumor recurrence in esophageal adenocarcinoma

**DOI:** 10.1038/s41598-018-32282-9

**Published:** 2018-09-18

**Authors:** Jianchun Gu, Jinhua Zhang, Leizhen Zheng, Jaffer A. Ajani, Xifeng Wu, Yuanqing Ye

**Affiliations:** 1 Departments of Epidemiology, The University of Texas MD Anderson Cancer Center, Houston, Texas USA; 2 Department of Oncology, Xinhua Hospital Affiliated to Shanghai Jiaotong University School of Medicine, Shanghai, China; 3 College of Life Sciences and Bioengineering, School of Science, Beijing Jiaotong University, Beijing, China; 4 Departments of Gastrointestinal Medical Oncology, The University of Texas MD Anderson Cancer Center, Houston, Texas USA

## Abstract

MicroRNAs (miRNAs) may contribute to the initiation and progression of cancer. The role of circulating miRNAs as predictors of recurrence in esophageal adenocarcinoma (EAC) has not been extensively explored. Here we measured the expressions of 167 miRNAs in serum samples from a discovery cohort of 72 EAC patients (32 patients with recurrence and 40 patients without). A rank sum test was performed to identify differentially expressed miRNAs. Cox regression model was applied to estimate the effect of miRNA expression on recurrence-free survival. The eligible miRNAs were then validated in an independent cohort of 329 EAC patients (132 patients with recurrence and 197 patients without). miR-331-3p was identified and confirmed to be differentially expressed between EAC patients with and without recurrence and associated with recurrence-free survival. In both cohorts, the expression of miR-331-3p was consistently decreased in patients with recurrence compared to those without (*P* < 0.05). Using patients with low expression of miR-331-3p as reference, those with high expression had HRs for recurrence of 0.45 (95% CI, 0.21-0.96, *P* = 0.040) and 0.55 (95% CI, 0.38–0.78, *P* = 0.001) in the discovery and validation cohorts, respectively. Therefore, serum miR-331–3p may be a useful biomarker for identifying EAC patients at high risk of recurrence.

## Introduction

Esophageal adenocarcinoma (EAC) is a fatal disease that is increasing in incidence^1^ and that currently accounts for over 60% of the new esophageal cancer cases in the United States^2^. EAC is usually diagnosed at an advanced-stage and treated with concurrent chemoradiotherapy followed by surgery in the United States, whereas chemotherapy alone before surgery is commonly used in Europe^3^. About 40–50% of EAC patients will develop a disease recurrence regardless of which type of treatment regimen is selected^4,5^. Identifying of EAC patients at high risk of recurrence is expected to contribute significantly to the implementation of personalized medicine, but current prognostic indicators, such as tumor stage and grade, have limited utility in the identification of high-risk patients. Therefore, new biomarkers for predicting EAC recurrence, especially those based on non-invasive or minimally invasive methods, are needed.

MicroRNAs (miRNAs) are small non-coding RNAs at a length of 18–25 nucleotides^6^. They are very powerful regulators capable of simultaneously influencing the expression of hundreds of genes by binding to the target messenger RNAs (mRNAs), resulting in either mRNA degradation or translation inhibition^7^. MiRNAs are involved in the regulation of multiple biological and pathological processes, and their aberrant expression contributes to the initiation and progression of cancer^8^. In recent years, miRNAs have emerged as promising biomarkers for cancer diagnosis^9^, prognosis^10^, recurrence^11^, and response to treatment^12^. A number of studies have investigated the role of circulating miRNAs as prognostic indicators in EAC^13^ and esophageal squamous cell carcinoma^14–16^. Additionally, specific circulating miRNAs, such as miR-21, miR-143, and miR-145, were found to be associated with higher risk of recurrence in esophageal squamous cell carcinoma^17,18^. However, few research has been done to identify potential miRNA markers for predicting tumor recurrence in EAC.

In this study, we screened for serum miRNAs that are differentially expressed in EAC patients with and without recurrence, and investigated their association with recurrence-free survival. Our aim was to explore the potential use of serum miRNAs as biomarkers for EAC recurrence.

## Results

### Patient characteristics

This study enrolled 401 EAC patients with a median follow-up time of 57 months (range: 7.6 to 127.5 months). The majority of the patients (92.5%) were male, which was consistent with male predominance in EAC. All patients received curative-intent therapy, including 225 patients received neoadjuvant chemoradiotherapy followed by surgery, 92 patients treated with concurrent chemoradiotherapy, and the remaining 84 patients underwent surgery only. The last patient without recurrence was followed up till 12 February 2014. At the time of analysis, 164 (40.9%) of the patients had experienced a recurrence. The median follow-up time was 61.9 months (95% CI, 55.4–65.4) for the recurrence-free patients. Patient’s clinical characteristics of each cohort were summarized in Table 1.

**Table 1:** Selected characteristics of the study population.

Characteristics	Discovery cohort	Validation cohort
	(N = 72)	(N = 329)
Age, mean (SD)	62.6 (4.2)	61.7 (10.9)
Gender, *n* (%)		
Male	71 (98.6)	300 (91.2)
Female	1 (1.4)	29 (8.8)
Smoking status		
Never	11 (15.3)	90 (27.4)
Former	43 (59.7)	158 (48.0)
Current	18 (25.0)	54 (16.4)
Unknown	0	27 (8.2)
BMI (kg/m^2^)		
<30	44 (61.1)	162 (49.2)
≥30	28 (38.9)	88 (26.8)
Unknown	0	79 (24.0)
Performance status		
0	30 (41.7)	172 (52.3)
1	26 (36.1)	115 (35.0)
2–4	2 (2.8)	5 (1.5)
Unknown	14 (19.4)	37 (11.2)
Stage, *n* (%)		
I	1 (1.4)	78 (23.7)
II	40 (55.5)	94 (28.5)
III	31 (43.1)	157 (47.7)
Grade, *n* (%)		
Well + moderately	38 (52.8)	162 (49.2)
Poorly	34 (47.2)	157 (47.7)
Unknown	0	10 (3.0)
Treatment regimen, *n* (%)		
nCRT + surgery	47 (65.3)	178 (54.1)
CRT	25 (34.7)	67 (20.4)
Surgery	0	84 (25.5)
Recurrence, *n* (%)		
No	40 (55.6)	197 (59.9)
Yes	32 (44.4)	132 (40.1)
MFT, months (range)	64.9 (26.1–104.6)	56.9 (7.6-237.2)

Abbreviations: CRT, chemoradiotherapy; nCRT: neoadjuvant chemoradiotherapy; MFT, median follow-up time.

### Discovery of miRNA markers

**Figure 1:**
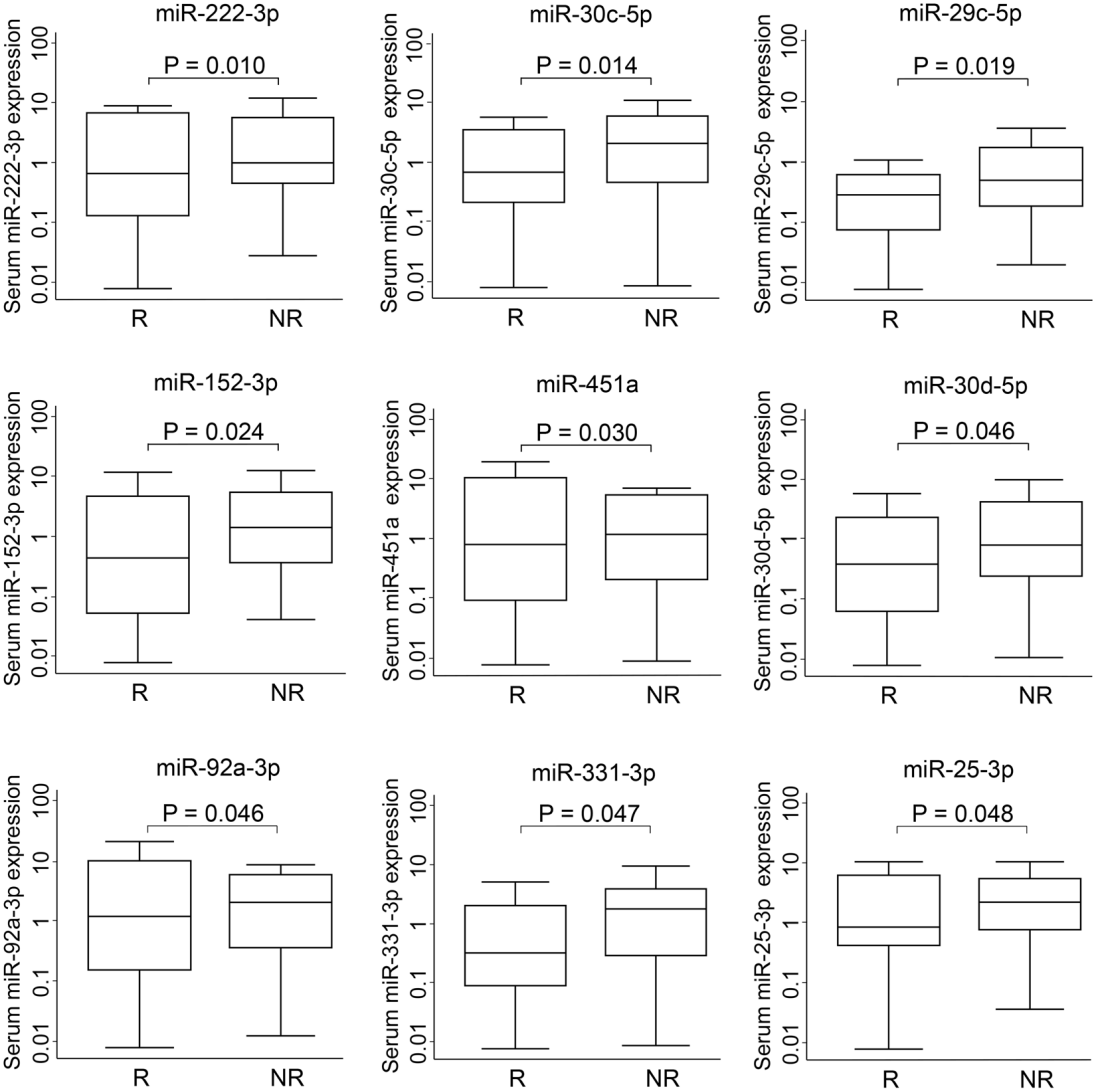
Expression levels of candidate miRNA biomarkers in the discovery phase. The Y axis shows expression levels on a log 10 scale. Levels of serum miR-222-3p, miR-30c-5p, miR-19b, miR-29c-5p, miR-152-3p, miR-451a, miR-30d-5p, miR-92a-3p, miR-331-3p, and miR-25-3p were compared between recurrent (R) and non-recurrent (NR) patients with esophageal adenocarcinoma. Statistical analyses were performed using a rank sum test.

Using the serum samples from the discovery cohort of 72 patients, we screened the expression levels of 167 serum miRNAs, as listed in Supplementary Table S1. Of these miRNAs, 107 (64.1%) were detected in at least 80% of samples and subjected to further analyses. To identify potential markers for predicting tumor recurrence, we selected candidate miRNAs that satisfied two criteria: (1) they were differentially expressed between patients with and without recurrence (*P* < 0.05, rank sum test), and (2) their expression levels were associated with recurrence-free survival (*P* < 0.05, Cox regression model, adjusted for patient age at diagnosis, gender, tumor grade, clinical stage, and treatment). Using the rank sum test, we identified nine miRNAs that were differentially expressed between the recurrence and non-recurrence groups (Fig. 1). A survival analysis using a Cox regression model demonstrated that four of these nine miRNAs (miR-25-3p, miR-152-3p, miR-331-3p, and miR-30c-5p) were significantly associated with recurrence-free survival (Table 2). All four miRNAs exhibited decreased expression in patients with recurrence compared to those without.

**Table 2:** Association of selected miRNAs with recurrence-free survival in the discovery and validation cohorts.

miRNA	Discovery cohort		Validation cohort		Meta-analysis		
	HR (95%CI)^a^	*P* value	HR (95%CI)^a^	*P* value	HR (95%CI)	*P* value	*P*-het
miR-152-3p (high vs low expression)	0.29 (0.12–0.68)	**0.005**	0.78 (0.54–1.11)	0.161	0.52 (0.20–1.33)^b^	0.169	0.038
miR-30c-5p (high vs low expression)	0.36 (0.16–0.82)	**0.015**	0.86 (0.61–1.22)	0.397	0.75 (0.55–1.04)^c^	0.082	0.057
miR-331-3p (high vs low expression)	0.45 (0.21–0.96)	**0.040**	0.55 (0.38–0.78)	**0.001**	0.53 (0.38–0.73)^c^	**<0.001**	0.652
miR-25-3p (high vs low expression)	0.44 (0.20–0.98)	**0.044**	1.04 (0.73–1.48)	0.817	0.91 (0.66–1.25)^c^	0.549	0.053

Abbreviations: CI, confidence interval; P-het, P for heterogeneity. Significant P values are indicated in bold font. ^a^Cox proportional hazards regression model, adjusted for age, gender, tumor grade, clinical stage, and treatment. ^b^Meta-analysis based on random effect model. ^c^Meta-analysis based on fixed effect model.

### Validation of miRNA markers

**Figure 2:**
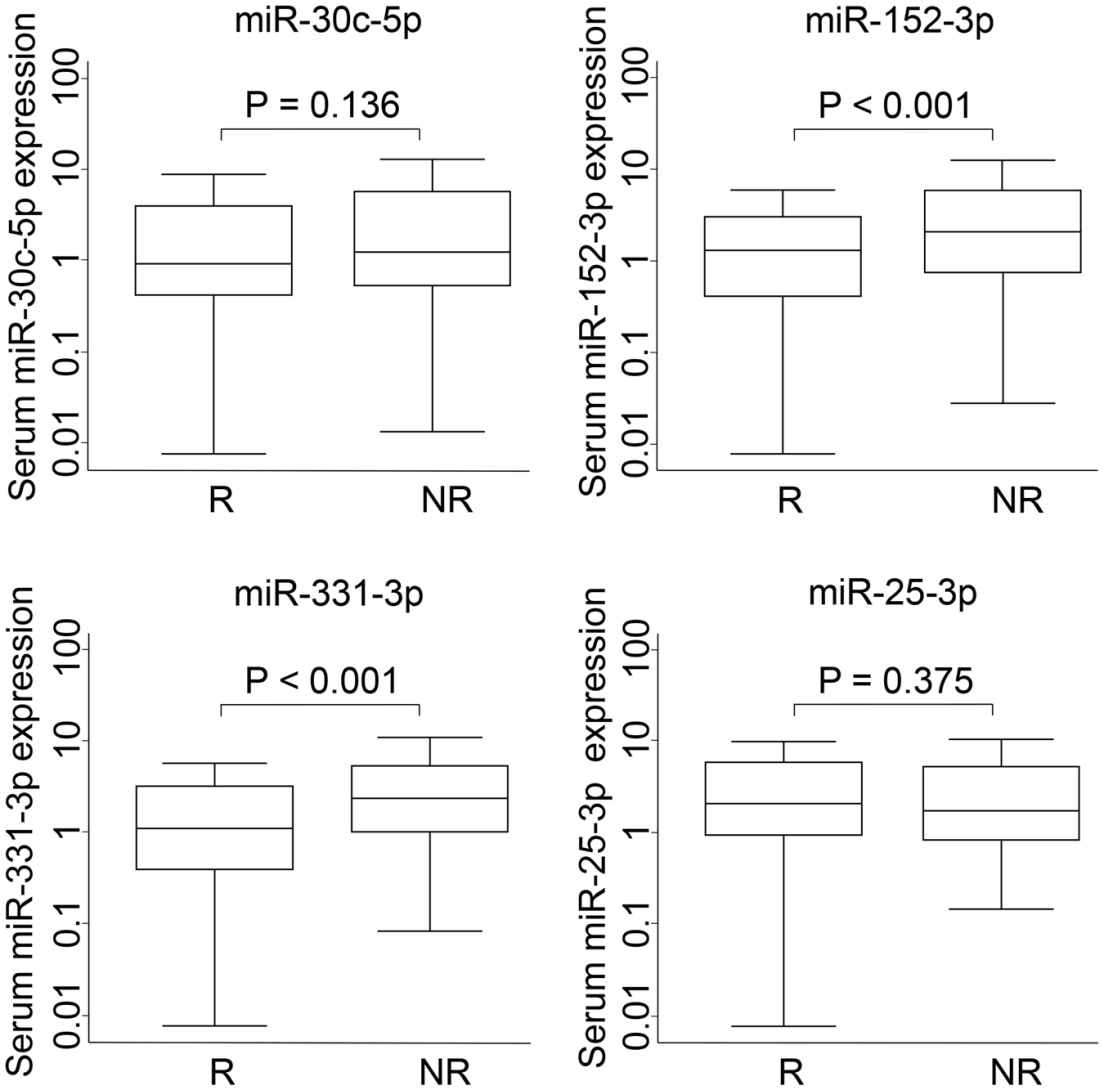
Expression levels of miR-30c-5p, miR-152-3p, miR-331-3p, and miR-25-3p in the validation phase. The Y axis shows expression levels on a log 10 scale. Levels of serum miRNAs were compared between recurrent (R) and non-recurrent (NR) patients with esophageal adenocarcinoma. A rank sum test was performed to compare groups.

**Figure 3:**
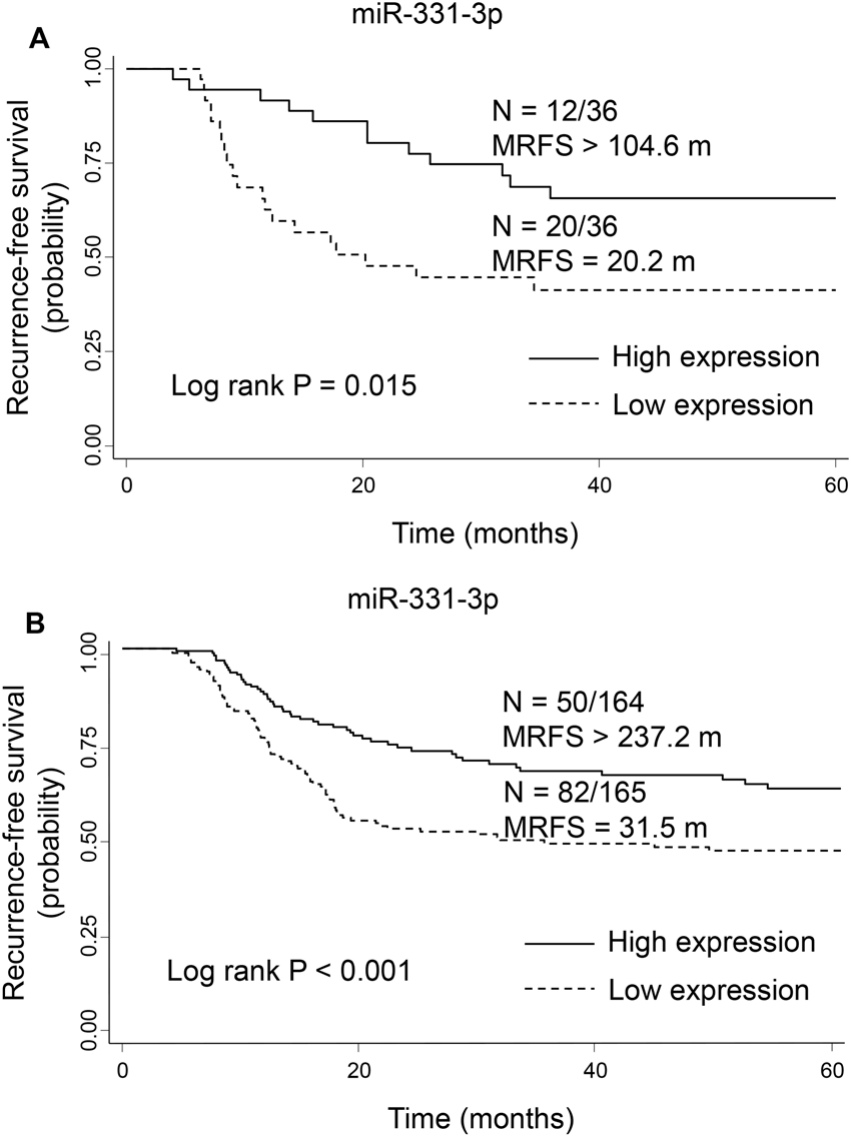
Kaplan-Meier curves for recurrence-free survival in the discovery (**A**) and validation (**B**) cohorts grouped by low and high levels of serum miR-331-3p. N = number of patients with an event (recurrence)/total number of patients in the dataset. MRFS, median recurrence-free survival time in months.

Next, the four miRNAs that showed the highest predictive value for recurrence in the discovery phase were measured in the validation cohort of 329 patients. We confirmed that two of the four, miR-152-3p and miR-331-3p, were significantly down-regulated in patients with recurrence compared with those without (Fig. 2). Furthermore, the expression level of miR-331-3p was significantly associated with recurrence-free survival, whereas the expression levels of the other three miRNAs were not (Table 2). Using patients with a low level of serum miR-331-3p as a reference, patients with a high level of miR-331-3p had HRs for recurrence of 0.45 (95% CI, 0.21-0.96; *P* = 0.040) and 0.55 (95% CI, 0.38–0.78; *P* = 0.001) in the discovery and validation cohorts, respectively. Meta-analysis of the association of miR-331-3p with recurrence-free survival under the fixed effect model showed a *P* value less than 0.001 (HR = 0.53; 95% CI, 0.38–0.73; *P* for heterogeneity = 0.65; Table 2). A Kaplan-Meier curve analysis showed that patients with a high expression of miR-331-3p had a significantly longer median recurrence-free survival time than those with a low expression (>237.2 months versus 31.5 months, log rank *P* < 0.001), which was consistent with the results for the discovery cohort (Fig. 3).

Nearly all of the patients (71/72, 98.6%) in the discovery cohort were diagnosed with stage II and III EAC, whereas 76.3% of patients in the validation cohort were classified as stage II and III. To address this imbalance, we analyzed the association of miR-331-3p with recurrence among the 251 patients with stage II and III disease in the validation cohort. The results showed that the stage II and III patients with a high level of miR-331-3p had a HR for recurrence of 0.52 (95%CI, 0.36–0.75, *P* = 0.001) compared to those with a low level of miR-331-3p, which were consistent with the pattern observed in the whole cohort.

## Discussion

Circulating miRNAs have several advantages as biomarkers, such as stability, abundance, and minimal invasiveness^19^, and emerging evidence indicates that they are promising prognostic indicators in patients with cancer^20^. Our study was one of the largest miRNA-related studies in EAC to date, focusing on the role of circulating miRNAs in predicting disease recurrence. A two stage study design was used with the discovery phase of 72 EAC patients followed by the validation phase of 329 EAC patients, aiming to minimize the potential of false positive findings. We found consistent and significant association of serum levels of miR-331-3p with risk of recurrence in both discovery and validation cohorts. Using patients with a low level of serum miR-331-3p as a reference, those with a high level of miR-331-3p had 55% and 45% reduced risk of recurrence in the discovery and validation cohorts, respectively. To our knowledge, this is the first report that serum miR-331-3p can stratify EAC patients at high risk of recurrence.

MiR-331-3p is a member of the miR-331 family and has been implicated as a tumor-suppressor. The expression levels of miR-331-3p were found to be significantly down-regulated in several malignancies, such as prostate cancer^21^, glioblastoma^22^, gastric cancer^23^, and colorectal cancer^24^. Here, in our study, miR-331-3p was shown to be significantly down-regulated in EAC patients with recurrence compared with those without. It has been demonstrated that overexpression of miR-331-3p inhibited the proliferation and migration of various cancer cells, suggesting the role of miR-331-3p as a tumor suppressor^22–26^. Functional data provided evidence that miR-331-3p could downregulate the expression of human epidermal growth factor receptor 2 (HER2). Two specific miR-331-3p target sites have been identified within the 3′-untranslated region of HER2 mRNA, and transfection of miR-331-3p in cancer cells reduced the expression of HER2 and inhibited its downstream PI3K/AKT pathway^21,24^. It is well-established that overexpression of HER2 and activation of its downstream pathways promote cancer cell proliferation, resistance to apoptosis, angiogenesis, and metastasis^27^. HER2 overexpression was detected in a variety of cancers and correlated with a poorer prognosis^28^, including EAC^29^. Therefore, the association of high miR-331-3p level with reduced risk of EAC recurrence identified in our study is possibly related to the role of miR-331-3p in downregulating HER2. It should be noted that some other mechanism might be involved. Wang *et al*. reported that miR-331-3p was significantly correlated with the cell cycle-related genes^30^. Transfection of miR-331-3p in gastric cancer cells induced G0/G1 phase cell cycle arrest by attenuating the expression of its direct target E2F1, which is crucial for G1/S transition^23^. In addition to downregulating the expression of HER2 and E2F1, miR-331-3p has been found to act as a tumor suppressor through regulation of neuropilin-2 (NRP2)^22,26^ and deoxyhypusine hydroxylase (DOHH)^31^. Further studies is needed to elucidate the concrete mechanisms for the effect of miR-331-3p expression on EAC recurrence.

However, the prognostic significance of miR-331-3p has not been well-documented. A recent study in 67 patients with hepatocellular carcinoma indicated that high levels of miR-331-3p was associated with a poor prognosis^32^. Potential reasons for the discrepancy between their results and ours include the different cancer sites^33,34^ and our study’s larger sample size. Future investigation is required to confirm our findings and to determine whether miR-331-3p has prognostic significance in other HER2-overexpressing cancers, such as breast cancer and gastric cancer.

One limitation is that our study did not randomize patients into discovery and validation cohorts. Most of the patients (98.6%) included in the discovery cohort were diagnosed with stage II and III EAC, whereas in the validation cohort 76.3% of patients were classified as stage II and III. To address this issue, we analyzed the association of miR-331-3p with recurrence in validation cohort patients with stage II and III disease, and the results were consistent with those from the whole cohort.

In conclusion, our study demonstrated for the first time that serum miR-331-3p was a potential biomarker for predicting tumor recurrence in patients with EAC. Prospective validation in independent external cohorts is necessary to confirm our findings, and further biological research is needed to clarify the underlying mechanisms.

## Patients and Methods

### Study Population

Our study included 401 white patients with newly diagnosed and histologically confirmed EAC who were recruited at The University of Texas MD Anderson Cancer Center during the period from 2003 through 2013. Patients were staged according to the 6th edition of American Joint Committee on cancer staging system, and patients with stage IV disease at diagnosis were excluded from the study. Serum samples of 72 patients (32 patients with recurrence and 40 patients without) were examined in the discovery phase. An independent sample set, consisting of serum samples from 132 patients with recurrence and 197 patients without, was used for validation. Smoking status and body mass index (BMI) were obtained from the baseline questionnaire during patients’ initial visit to MD Anderson Cancer Center. Clinical and follow-up data were abstracted by medical chart review. This study was approved by the MD Anderson Cancer Center’s Institutional Review Board, and all methods were performed in accordance to the relevant guidelines and regulations as described in the institutional policies and the study protocol. All participants have provided informed written consent.

### Quantification of miRNAs by Fluidigm arrays

Whole blood samples were collected from each patient before treatment and immediately separated into serum, which was stored at liquid nitrogen tanks until the assays were performed. Total RNA was isolated from 750 μl serum samples using miRNeasy Mini Kit (Qiagen). The concentration and purity of the RNA were evaluated using a NanoDrop ND-100 spectrophotometer (Thermal Scientific). Purified RNA samples were reverse transcribed with TaqMan miRNA Reverse Transcription Kit (Applied Biosystems), using 150 ng RNA and pools of Megaplex RT Primers, followed by a pre-amplification step with Megaplex PreAmp Primers. Real-time PCR quantification using Fluidigm 96.96 dynamic arrays and Biomark HD detection system (Fluidigm Corp, San Francisco, CA) were employed to measure serum miRNA expression in both discovery and validation cohorts according to the manufacturer’s protocol. The expression levels of the miRNAs were normalized to spiked-in cel-miR-39 and cel-miR-54, and the 2^−ΔΔCt^ method was used for analysis^35,36^. A total of 167 miRNAs, selected according to literature data, were measured in our discovery set. MiRNAs detected in at least 80% of the serum samples were further analyzed. The miRNAs that were differentially expressed between recurrent and non-recurrent patients were assessed for the association with recurrence-free survival. The most significant miRNAs were then evaluated in the validation set.

### Statistical analysis

A rank sum test was applied to evaluate the differences in median miRNA expression levels between patients with and without recurrence. The associations of miRNA expression levels with recurrence-free survival were estimated as hazard ratios (HRs) and its 95% confidence interval (CI), using Cox proportional hazards regression model, adjusted for age (continuous), gender (male or female), tumor grade (well, moderately, or poorly), clinical stage (I, II, or III), and treatment (surgery, chemoradiotherapy, or neoadjuvant chemoradiotherapy plus surgery). The proportional hazards assumption was checked to be valid by inspecting the log-log survival curves visually and testing the significance of the interaction term between time and the covariate. Recurrence-free survival time was defined as the time in months between the date of diagnosis and the date of first event of either recurrence or death. There was no cutoff point used for subject follow-up to categorize patients with and without recurrence. All patients were followed until their last date of follow-up or until the date of death. Kaplan-Meier method was used to estimate recurrence-free survival, and the significance was determined by log-rank test. Serum miRNA expression was classified as high or low using the median value as the cut-off. A meta-analysis was performed to combine the results from the discovery and validation cohorts. Heterogeneity was assessed using χ^2^-based Q-statistics. A fixed effect model was used when heterogeneity was not detected (*P* for heterogeneity > 0.05); otherwise, a random effect model was adopted. Statistical analyses were performed using STATA software 14.0 (Stata Corp, College Station, TX). All tests were two-sided, and a *P* value less than 0.05 was considered statistically significant.

## Electronic supplementary material

Supplementary Table S1

## Data Availability

Analyzed data generated from this study are available to the readers upon publication pending corresponding author’s approval. Data containing protected patient identification and personal information will be removed. Materials and protocols are available pending institutional approval via collaboration and materials transfer agreement.
