# Supplementary material for: Serum miR-331-3p predicts tumor recurrence in esophageal adenocarcinoma
Source: Sci Rep. 2018 Sep 18;8:14006. doi: 10.1038/s41598-018-32282-9 (PMC6143616; doi:10.1038/s41598-018-32282-9)
Supplement: Supplementary file 1 — Supplementary Table S1 [file 41598_2018_32282_MOESM1_ESM.docx]

**Serum miR-331-3p predicts tumor recurrence in esophageal adenocarcinoma**

Jianchun Gu^1, 2^, Jinhua Zhang^1, 3^, Leizhen Zheng^2^[, Jaffer A. Ajani](http://www.ncbi.nlm.nih.gov/pubmed/?term=Ajani%20JA%5BAuthor%5D&cauthor=true&cauthor_uid=25998788)^4^, Xifeng [Wu](http://www.ncbi.nlm.nih.gov/pubmed/?term=Wu%20X%5BAuthor%5D&cauthor=true&cauthor_uid=25998788)^1^*, and Yuanqing [Ye](http://www.ncbi.nlm.nih.gov/pubmed/?term=Ye%20Y%5BAuthor%5D&cauthor=true&cauthor_uid=25998788)^1^

Departments of ^1^Epidemiology and ^4^Gastrointestinal Medical Oncology, The University of Texas MD Anderson Cancer Center, Houston, Texas, USA;

^2^Department of Oncology, Xinhua Hospital Affiliated to Shanghai Jiaotong University School of Medicine, Shanghai, China;

^3^College of Life Sciences and Bioengineering, School of Science, Beijing Jiaotong University, Beijing, China.

**Supplementary Table S1**. The 167 serum miRNAs measured in the discovery cohort

| let-7a | miR-103 | miR-193a-5P | miR-375 |
| --- | --- | --- | --- |
| let-7b | miR-106a | miR-193b | miR-376a |
| let-7c | miR-106b | miR-194 | miR-376c |
| let-7d | miR-107 | miR-195 | miR-378 |
| let-7e | miR-125a-3P | miR-197 | miR-381 |
| let-7g | miR-125a-5P | miR-199a-3P | miR-409-3P |
| miR-10a | miR-125b | miR-199a-5P | miR-410 |
| miR-10b | miR-126 | miR-199b | miR-422a |
| miR-15a | miR-127-3P | miR-200c | miR-423-5P |
| miR-15b | miR-128 | miR-203 | miR-425 |
| miR-16 | miR-1290 | miR-206 | miR-432 |
| miR-17-3P | miR-1291 | miR-210 | miR-433 |
| miR-18a | miR-130a | miR-211 | miR-451a |
| miR-19a | miR-130b | miR-212 | miR-454 |
| miR-19b | miR-132 | miR-215 | miR-483-5P |
| miR-20a | miR-134 | miR-218 | miR-484 |
| miR-20b | miR-139-3P | miR-221 | miR-486-5P |
| miR-21 | miR-139-5P | miR-222-3p | miR-491-5P |
| miR-22 | miR-140-3P | miR-223 | miR-495 |
| miR-23a | miR-140-5P | miR-296-5P | miR-505-3P |
| miR-24 | miR-142-3P | miR-301a | miR-532-3P |
| miR-25-3p | miR-142-5P | miR-302c | miR-532-5P |
| miR-26a | miR-143 | miR-320 | miR-574-3P |
| miR-26b | miR-144 | miR-320b | miR-590-5P |
| miR-27a | miR-145 | miR-323-3P | miR-598 |
| miR-27b | miR-146a | miR-324-3P | miR-625 |
| miR-28-3P | miR-146b-3P | miR-324-5P | miR-628-3P |
| miR-29a | miR-146b-5P | miR-328 | miR-629 |
| miR-29b | miR-148a | miR-331-3P | miR-642 |
| miR-29c-5p | miR-148b | miR-335 | miR-652 |
| miR-30a-3P | miR-150 | miR-339-3P | miR-664 |
| miR-30b | miR-151-3P | miR-339-5P | miR-671-3P |
| miR-30c-5p | miR-151-5P | miR-340 | miR-708 |
| miR-30d-5p | miR-152-3p | miR-342-3P | miR-720 |
| miR-30e-3P | miR-155 | miR-345 | miR-744 |
| miR-34a | miR-181a | miR-361-5P | miR-766 |
| miR-92a-3p | miR-183 | miR-363 | miR-769-5P |
| miR-93 | miR-185 | miR-365 | miR-886-5P |
| miR-99a | miR-186 | miR-367 | miR-942 |
| miR-99b | miR-190b | miR-370 | miR-1274a |
| miR-100 | miR-191 | miR-374a | miR-1274b |
| miR-101 | miR-192 | miR-374b |  |
